# Supplementary material for: Are carbon emissions trading and green financial instruments synergistic? -Comprehensive quantitative research based on content analysis
Source: PLoS One. 2024 Mar 7;19(3):e0298601. doi: 10.1371/journal.pone.0298601 (PMC10919601; doi:10.1371/journal.pone.0298601)
Supplement: S1 Data — (ZIP) [file pone.0298601.s001.zip › Data/地级市Z_F工作报告.zip!/╜¡╬≈/╕o╓▌╩╨/└┤╫╘╣1⁄2╓┌║┼í╛┬φ┐╦╩2╛▌═°í┐_2016.docx]

各位代表：

现在，我代表市人民政府向大会报告工作，请予审议，并请各位市政协委员和列席会议的同志提出意见。

一、2015年及“十二五”发展主要成效

刚刚过去的一年，是经济发展进入新常态的转折之年，也是“十二五”规划的收官之年。一年来，在省委、省政府和市委的坚强领导下，全市上下全面贯彻省委 “十六字”方针，按照市委“高位谋势、精准发力、敢于担当、跨越发展”的工作总要求，主动适应新常态，坚持求真务实、开拓创新，经济发展稳中有进、稳中向好，社会事业全面进步。全市实现生产总值1105.14亿元，增长9.2%;财政总收入165.2亿元，增长10.1%;公共财政预算收入126.82亿元，增长9%;固定资产投资1099.67亿元，增长17.1%;规模以上工业增加值364.23亿元，增长8.6%;社会消费品零售总额428.2亿元，增长9.4%;出口总额102.28亿元，增长8.1%;实际利用外资2.93亿美元，增长16.8%;城镇居民和农村居民人均可支配收入分别达到25065元和11441元，增长8.5%和9.9%;居民消费价格总体水平上升0.9%;城镇登记失业率控制在3.3%以内。

一年来，我们主要抓了以下六方面工作：

(一)全力应对经济下行压力，着力稳定经济增长。加大对实体经济的帮扶力度。在落实好国家、省稳增长政策的基础上，注重打好我市政策“组合拳”。制定出台了稳增长促升级50条、加快抚州国家高新区发展升级22条、促进房地产市场平稳健康发展12条等政策措施。建立了覆盖全市规上工业企业的市、县(区) 领导挂点帮扶机制，派遣了“企业特派员”。建立了全市经济运行分析监测制度，在全省率先建立工业运行监测分析二级平台。加强重大平台建设。抚州高新技术产业园区升级为国家高新技术产业开发区，抚州国家高新区、向莆经济带、赣闽产业合作示范区、抚州市国家中小学教育质量综合评价改革实验区(省级基础教育综合改革试验区)列入省原中央苏区振兴重点平台。加快推进一批重大项目。大唐抚州电厂一期工程1×100万千瓦机组投产发电，500千伏抚州电厂送出线路工程、500千伏抚州2号主变扩建工程竣工投产，电网建设投资创历史新高;抚金高速、昌宁高速(乐安段)建成通车;省天然气管网一期工程抚州—南城—黎川支线工程建成投运;资光、船广、东昌、广吉高速和500千伏抚州至红都输电线路工程等项目有序推进;东外环(抚金高速与东昌高速连接线)纳入省“十二五”高速路网规划并完成设计;鹰梅铁路和吉抚武铁路项目初步纳入国家中长期铁路网调整规划;廖坊水利枢纽灌区二期工程可研报告获省发改委批复;昌抚城际铁路列入省“十三五”重大项目。着力稳定传统消费，扩大新型消费。商品房销售面积增幅持续扩大，批发零售业、住宿餐饮业消费逐步回暖。不断壮大外贸实体，加强重点出口企业帮扶。全市新增出口生产企业30家，达153家，占出口企业总数70.8%。

强实体企业金融服务。进一步扩大“财园信贷通”、“财政惠农信贷通”、“税贷通”等政策扶持范围;在全省率先推行过桥贷款制度，设立了2.61亿元企业融资转贷基金，建立了银行抽贷报告制度和问题企业贷款协调处置制度，发放小额贷款9.83亿元，有力地支持了实体经济发展。

(二)注重经济提质增效，推动产业发展升级。做强做大工业经济。加快产业集聚发展。电子信息、生物医药、食品加工、机电汽车四大产业分别完成主营业务收入36.6亿元、66亿元、113.7亿元、273.7亿元，增长35.7%、8.7%、9.9%和15.1%;崇仁工业园区成功创建“国家新型工业化产业示范基地”，金溪香精香料产业被确定为“全国林木香料生物产业基地”。一批重大工业项目加速推进。投资13.5亿元的博雅欣和制药、投资30亿元的森鸿科技、投资50亿元的广东海利不锈钢管及磨砂板、投资15亿元的广药集团食品饮料等项目加快建设，广银铝业一期 3.6万吨铝型材项目试生产。世界第二大钛白粉企业沙特科斯特公司成功收购添光化工。自立环保二期完成拆迁7.3万m2，启动了深加工项目建设，为企业上百亿台阶奠定基础。推动企业创新发展。新增4个省级工程技术中心、1个省重点实验室和2个省级产业技术创新战略联盟;新增认定23家高新技术企业，增长55.2%;高新技术产业产值和增加值分别增长21.83%和22.37%。博雅生物设立全市首个博士后工作站。加快建设现代农业强市。粮食生产实现“十二连丰”;现代农业示范园区建设快速推进，编制完成并向国家申报了全市域国家现代农业示范区规划，11个县(区)示范园共投入47.06亿元，核心区面积达19.8万亩，入园项目实现产值130亿元;十大农业产业集群完成销售收入177.5亿元，增长10%;农产品综合加工转化率达到46%，比上年提高2个百分点。投资10亿元加强了农田水利基础设施建设。东乡、金溪被认定为省级现代农业示范区，东乡润邦获批全省唯一的国家首批区域生态循环农业示范点;苍源药业成为全省第一家在“新三板”挂牌的农业企业。加快发展现代服务业。资溪大觉山景区入选国家5A级景区预备名单，南丰橘文化旅游产业集聚区成功创建国家4A级景区，全市旅游接待总人数、旅游总收入增长28%和38%。江西银行、赣州银行、兴业银行在抚分支机构开业;金融机构贷款余额比年初增加183.76亿元，增长24.3%，增速全省第一;成功发行8亿元中期票据，债务融资工具实现零的突破;新增3家企业在“新三板”挂牌，7家企业在上海股权托管交易中心Q板或E板挂牌。建筑业完成产值293.65亿元、增长16.8%，实现税收25.7亿元、增长22%，新增1家特级企业和9家一级企业。抚州综合物流产业集群和南丰蜜橘物流产业集群纳入全省16个重点物流产业集群。电子商务交易额达200亿元，比上年翻了一番。华美立家建材家居广场建成开业，大润发、华润万家超市进驻市中心城区，万达商业中心建设加快推进。

(三)深入推进改革开放，深化区域合作发展。强力推进重点领域改革。加大简政放权力度，对国务院、省政府取消下放的219项行政审批事项全部承接到位，市本级行政权力精简67.6%，取消非行政许可审批事项;“三单一网”快速推进，公布了市级行政权力清单和责任清单。全面实施“三证合一”、“一照一码”，审批时限大幅缩减。市、县机构改革任务全面完成。基本完成农村土地承包经营权确权登记颁证，启动市级农村综合产权交易市场建设、扩权强镇试点和农村集体产权制度改革试点。推进市投资发展公司实体化转型改革，组建了市工创投和市旅投公司。推动临川教育集团学校转型升级，引进社会资本兴教办学，抚州教育品牌更加响亮。积极主动深化区域合作。加快融入南昌。实现了昌抚通讯、金融、医疗、社保、公交、公积金等同城化;《昌抚合作示范区规划》获省发改委批准，昌抚产业合作开始起步。主动对接海西。与莆田市签订了跨区域口岸合作框架协议;编制完成了《赣闽台产业合作示范区规划》，正待省政府审批;扎实推进抚州海西综合物流园建设，一批物流集聚区加快建设。加强与南昌和海西地区的经贸合作交流。成立了市台商协会;与向莆铁路沿线城市签署了旅游合作协议，并在多个领域进行了深入广泛合作。与闽浙赣、闽粤赣、闽浙赣皖区域合作组织联系对接进一步加强。进一步扩大对外开放。在全国先后举办18场专题招商活动，签约项目178个、总额1121.44亿元;实际利用外资2.93亿美元、增长16.8%，其中现汇进资1亿美元，两项增幅均为全省第一。与江西对外经济合作公司签订战略合作协议，中阳建设参与“一带一路”建设开发，赣东路桥、志特科技、嘉盛精纺开拓海外市场，企业“走出去”取得突破。

(四)扎实推进新型城镇化，建管水平稳步提升。着眼完善功能、补齐短板，在市中心城区实施了赣东大道路面改造、金巢大道南延伸段、文昌大道拓宽改造、汝水南大道、科技大道等一批城市道路基础设施项目，推进了抚州一中改扩建、市第三医院、市第一人民医院老院区门急诊楼建设等一批社会事业项目，保障性住房、棚户区改造、老城区市政基础设施改造等一批民生项目加快推进，特别是在4个月内，基本完成了全省规模最大、保存最为完整的历史文化街区—文昌里棚户区改造项目国有土地上房屋征迁工作。总部经济中心、赣东国际汽车城等一批城市产业项目进展顺利。以创建省级文明城市活动为载体，铁心硬手整治城市环境，在市中心城区集中开展了“五车”整治、交通秩序整治、环境卫生综合整治，城市秩序明显好转，市容环境明显改善。东乡、临川乡镇综合行政执法改革试点进展良好。深入开展国省干线沿线乡镇环境综合整治，取得良好成效。中心村整治建设和农村生活垃圾专项治理工作纵深推进。

(五)致力提升生态优势，加快生态文明建设步伐。守住生态环境安全底线，划定生态保护红线总面积5755.8平方公里，占全市区划面积的30.6%。强化以工业废气、机动车尾气和城市扬尘污染为重点的“净空”治理行动。启动了PM2.5监测并与国家联网，空气质量保持全省前列。强化以抚河、宜黄河、崇仁河水系区域工业、生活污水排放治理为重点的“净水”行动。建立了“河长制”，基本完成了临川、南丰、南城、金溪、宜黄5个县(区)工业园区污水处理厂及配套管网建设，12个全省百强中心镇污水处理设施和配套管网工程建设全面开工;12个地表水监测断面和集中式饮用水源地水质达标率均为100%。强化以城乡生活垃圾专项治理、农村面源污染、重金属污染治理为重点的“净土”行动。推进了以清洁家园、清洁水源、清洁田园为主要内容的“清洁工程”建设;在全省率先实施全市域封山育林;洪门水库列入全国生态湖泊治理项目;新创建2个国家级生态文化村、1个省级生态乡镇和5个省级生态村。

(六)着力增进民生福祉，提高人民群众获得感。将新增财力向民生倾斜，民生支出增长14.7%。全面完成了省政府下达的50件民生实事。扎实推进精准扶贫。全面完成了253个贫困村的扶贫对象建档立卡工作，通过产业扶贫、移民搬迁、保障兜底，实现了5万人精准脱贫目标。就业形势保持稳定。城镇新增就业 4.39万人，零就业家庭就业继续保持动态清零。切实加强民生保障。城乡低保、农村五保、残疾人、城乡居民养老保险待遇、企业退休人员基本养老金等保障水平稳步提高;完成城镇保障性安居工程12153套，新建、改造农村危房20144户。不断发展社会事业。市直学校实现“零择校”，教育质量公平明显提升;医药卫生体制改革深入推进，公共卫生服务水平持续提高;全面启动汤显祖逝世400周年纪念活动筹备工作，临川文化品牌影响力进一步扩大。军民融合深度发展。成功组织“红土地2015”国防动员演习，国防动员、民兵预备役、人民防空等各项建设扎实推进。信访形势明显好转，安全生产和治安形势持续向好，社会大局保持稳定。体育、卫生和人口计划生育、食品药品监督、国资监管、市场与质量监管、行政司法、民族宗教、外事侨务、涉台事务、审计、粮食、统计调查、防震减灾、气象、水文、档案、地方志、妇女儿童、老龄、残联、红十字会、驻外办事处等工作有了新的进步。

一年来，我们注重加强政府自身建设，促进工作作风转变。开展“三严三实”专题教育，狠抓问题整改和机制健全完善。建立了法治政府建设指标体系。认真执行市政府常务会议学法制度，严格落实重大行政决策制度，认真办理市人大代表建议和市政协提案，主动接受依法监督和民主监督。加大抓落实工作力度，健全完善了重大工程、重点项目定期调度、跟踪督查机制。加强政府系统党风廉政建设和反腐败工作，强化行政监察、财政监管、审计监督，查处了一批违法违纪典型案件。

需要如实向各位代表报告的是，在经济发展进入新常态、增长速度由高速向中高速转变的大背景下，全市经济运行面临前所未有的下行压力，2015年部分经济指标低于年初计划目标。对照“十二五”规划要求，有部分经济指标略低于预期。但总体上看，我市“十二五”规划主要目标任务基本完成，抚州发展站到了新的历史起点上。经过五年的发展，全市综合实力持续增强，发展质量不断提升。生产总值、固定资产投资突破千亿元大关。财政总收入、公共财政预算收入实现四年翻番。生产总值和财政总收入在全省排名双双前移1位。工业税收成为第一大税源。财政收入超10亿元县(区)由1个增至8个，东乡、临川突破20亿元。重大基础设施建设加快推进，发展基础进一步夯实。争取到我市纳入原中央苏区振兴发展国家战略，支持抚州深化区域合作加快发展上升为省级战略。抚州人民的高速梦、高铁梦、电厂梦和汽车梦圆满实现。城镇化进程不断加快，城乡面貌深刻变化。市中心城区建成区面积从50.3平方公里扩大到63平方公里;成功创建“国家园林城市”、“国家森林城市”、“省级卫生城市”和“省级文明城市”。城镇化率提高7.8个百分点，达到45%。投入26.4亿元，完成了 3794个新农村建设村点整治建设。改革开放深入推进，发展活力明显增强。启动各类改革试点、改革示范及试验区建设56个，一批改革试点走在全省前列。引进5000万元以上市外境内项目1877个、资金1608亿元，直接利用外资、出口总额年均增长13.7%和26.95%。生态文明建设扎实推进，生态优势日益凸显。全市森林覆盖率达65.6%，绿地率达37.45%，绿化覆盖率达41.34%，万元GDP能耗下降13%。被中国标准化研究院评为 “2015年中国大陆城市‘氧吧’50强”第一名。社会事业全面发展，人民生活得到较大改善。“四条保障线”全面建立。新增就业24.9万人，贫困人口由 31.6万人下降到13.9万人。2015年城镇居民和农村居民人均可支配收入分别为2010年的1.73倍和1.9倍。建设城镇保障性住房45083 套、250.23万m2，改造农村危房57239户，发展成果更多惠及广大群众。

各位代表，发展历程饱含奋斗，发展成果来之不易。这是省委、省政府和市委正确领导的结果，也是全市上下团结奋进、扎实苦干的结果。在此，我代表市人民政府，向全市广大干部群众，向各民主党派、工商联、无党派、新阶层、人民团体、社会各界人士，向中央、省驻抚单位，驻抚部队和武警、消防官兵，向外来投资者和建设者，向所有关心、支持和帮助抚州发展的同志们、朋友们，致以崇高的敬意和衷心的感谢!

当前，我市经济社会发展还存在不少矛盾和问题。经济总量小、人均水平低的状况尚未得到根本改变;工业基础较为薄弱，产业技术含量不高，企业创新能力不强;现代农业发展不快，农产品加工转化率较低;消费需求不旺，服务业增长动能不足;涉及人民群众切身利益的住房保障、教育、医疗、养老等优质公共服务供给不足，存在短板;政府效能有待提升，少数地方和部门还存在不作为、慢作为、乱作为的现象。对于这些问题，我们将认真面对，坚决改进。
